# Evaluation of bloodstream infections in febrile neutropenic patients with hematological malignancy: a retrospective cohort study

**DOI:** 10.1186/s12879-026-13465-6

**Published:** 2026-04-29

**Authors:** Muhammet Salih Tarhan, Yusuf Emre Özdemir, Laden Çamber, Fehmi Hindilerden, Emine Gültürk, Habip Gedik, Kadriye Kart Yaşar

**Affiliations:** 1 Department of Infectious Diseases and Clinical Microbiology, Mardin Training and Research Hospital, Mardin, Türkiye; 2 Department of Infectious Diseases and Clinical Microbiology, Bakırköy Dr. Sadi Konuk Training and Research Hospital, İstanbul, Türkiye; 3 Department of Hematology, Bakırköy Dr. Sadi Konuk Training and Research Hospital, Istanbul, Türkiye

**Keywords:** Bacteremia, Febrile neutropenia, Hematological malignancy, Mortality

## Abstract

**Background:**

Patients with hematological malignancies (HMs) are at high risk of bloodstream infections (BSIs) during episodes of febrile neutropenia (FN). However, empirical treatment initiated before isolation of the causative agent from blood cultures is crucial. The aim of this study was to determine the distribution and antimicrobial resistance profile of microorganisms isolated from BSIs in patients with HMs, and to evaluate the association with the need for intensive care unit (ICU) and mortality at 30 days.

**Methods:**

Single-centre data from patients with HMs who were admitted to the hematology inpatient clinic and had a BSI during an episode of FN were retrospectively reviewed.

**Results:**

A total of 164 FN episodes with BSI in 122 patients were analysed. Of the 164 episodes, 56.7% were Gram-negative. The most prevalent Gram-negative bacterium was *Escherichia coli* (31%). The positivity rate for extended-spectrum beta-lactamase (ESBL) was 49.3%, and the rate of carbapenem resistance was 20.2%. In multivariate analyses restricted to Gram-negative BSIs, carbapenem resistance was independently associated with both 30-day ICU admission and 30-day all-cause mortality, whereas ESBL positivity was not independently associated.

**Conclusion:**

The rates of ESBL positivity and carbapenem resistance in Gram-negative bacteria are notable. However, only carbapenem resistance was independently associated with adverse clinical outcomes. Therefore, in line with regional surveillance data, empirical antibiotic strategies should take into account local resistance patterns, particularly carbapenem resistance, in FN episodes.

## Background

Neutropenia, a common condition in patients with hematological malignancies (HMs), has been demonstrated to raise the risk of infection. In addition to neutropenia, the chemotherapeutic agents administered to these patients also induce immunosuppression, thereby facilitating the development of infections [1]. Some of the chemotherapeutic agents administered to these patients have been observed to impair neutrophil function and induce mucositis in the gastrointestinal tract. Mucositis provides a gateway for microbial pathogens to gain access to the body. In these patients, the source of infection is frequently the patient’s own gastrointestinal tract [2]. Patients with HMs are at markedly increased risk of developing bloodstream infections (BSIs), with a 10-fold increased incidence compared to patients without malignancies [3]. In this patient group, BSIs have been associated with an increased risk of morbidity and mortality and a negative impact on quality of life [4]. The etiology of febrile neutropenia (FN) is frequently unknown at the initiation of treatment. The selection of empirical antimicrobial therapy should be based on the prevalence of pathogens and their susceptibility patterns in the region, the risk group (high or low risk for complications), and the potential origin of infection [5]. It is crucial to determine which microorganisms are most commonly responsible for BSIs and to characterize their antimicrobial resistance patterns. The objective of this study was to determine the causative agents of BSIs and their antimicrobial resistance profiles in FN episodes among patients with HMs, and to evaluate the relationship between these pathogens and the need for intensive care unit (ICU) admission and mortality rates at 30 days.

## Methods

This single-centre retrospective study was conducted at the Hematology Clinic of Istanbul Bakırköy Dr. Sadi Konuk Training and Research Hospital between September 2015 and March 2024. The study included patients aged 18 years and older who were hospitalised and followed due to HMs, had an episode of FN, and were diagnosed with BSI.

In order to exclude contamination, the number of culture bottles with growth, the clinical status of patient and laboratory values were taken into consideration when skin flora were isolated in the blood cultures. Following this, a decision was made on whether the isolated microorganism was the causative agent. Any growths deemed to be the result of contamination were excluded from the study. For each patient, the following data were collected: age, gender, presence of chronic disease, underlying HMs, number of blood cultures with growth, microorganisms grown, antibiotic resistance status of microorganisms and empirical antibiotic treatment. Furthermore, patients were assessed for the need for ICU admission and all-cause mortality within the initial 30-day period following the onset of FN.

### Definitions

FN episode was defined as a fever of 38.3 °C or higher that persists for at least one hour in patients with a neutrophil count below 500/mm^3^ or between 500 and 1000/mm^3^ and tends to fall below 500/mm^3^ within 48 h [6].

The number of neutropenic days was defined as the duration of neutropenia prior to the onset of each BSI episode. In patients with multiple BSI episodes, the number of neutropenic days was calculated separately for each episode.

BSI was defined in two ways. First, isolation of microorganisms, excluding skin flora, from at least one blood culture. Second, isolation of skin flora (e.g., diphtheroids, *Bacillus* spp., *Propionibacterium* spp., coagulase-negative staphylococci (CoNS), and micrococci) from two or more consecutive blood cultures with clinical evidence of infection. BSIs were classified as central venous catheter–associated BSI, mucosal barrier injury–laboratory confirmed bloodstream infection (MBI-LCBI), or secondary BSI with a documented source of infection, based on standard clinical and microbiological criteria. Catheter-related BSI was defined as a laboratory-confirmed BSI occurring in a patient with an intravascular catheter, with no other apparent source of infection, and isolation of the same microorganism from both catheter-drawn and peripheral blood cultures. MBI-LCBI was defined as a laboratory-confirmed BSI in neutropenic patients with hematologic malignancy, in the absence of another infection source, consistent with mucosal translocation of endogenous flora. Secondary BSI was defined as a laboratory-confirmed BSI occurring in association with a documented primary site of infection caused by the same microorganism. BSI episodes were considered distinct if they occurred during separate febrile neutropenia episodes and were separated by ≥ 14 days after clinical resolution, or if caused by a different microorganism.

ICU admission and mortality were defined as ICU transfer and 30-day all-cause mortality, occurring after the onset of FN.

### Microbiological diagnosis

The blood cultures were analysed using the BACTEC^®^ automated system (Becton Dickinson, Franklin Lakes, NJ, USA). Conventional diagnostic methods were applied for bacterial typing and antibiogram. When necessary, the VITEK^®^ 2 automated system (bioMerieux, Marcy l’Etoile, France) was used. Antibiotic susceptibility testing was performed on Mueller-Hinton agar using the Kirby-Bauer disc diffusion method, with the gradient diffusion method used when necessary. Extended-spectrum beta-lactamase (ESBL) production was documented based on automated susceptibility testing results (VITEK^®^ 2 system). Colistin susceptibility testing was selectively performed in Gram-negative isolates exhibiting ESBL production and/or carbapenem resistance, using a broth dilution–based method. Candida species were identified using VITEK^®^ 2, and antifungal susceptibility testing was performed using the same system. Antibiotic susceptibility results were evaluated according to the European Committee on Antimicrobial Susceptibility Testing (EUCAST) guidelines [7].

### Ethics

This study was approved by the Clinical Research Ethics Committee of Bakirkoy Dr. Sadi Konuk Training and Research Hospital (Approval No: 2024-02-01; Protocol Code: 2024/119). The study was conducted in accordance with the Declaration of Helsinki and ensured participants’ anonymity, confidentiality.

### Statistical analysis

The statistical analysis was conducted using the SPSS 27.0 software (Statistical Package for the Social Sciences). The research data was evaluated using descriptive statistical approaches, including mean, standard deviation, median, frequency, and ratio. The chi-square test was performed to compare nominal data; when the sample size was less than 15, Fisher’s exact test was used instead. Multivariate logistic regression analyses were performed to evaluate the independent association between antimicrobial resistance phenotypes and clinical outcomes (ICU admission and 30-day mortality). Adjusted odds ratios (ORs) with 95% confidence intervals (CIs) were reported. Statistical significance was defined as *p* < 0.05.

## Results

A total of 122 patients with FN were evaluated in this study, of whom 66 were male (54.1%). The mean age of the patients was 50.11 ± 16.35 years. The most common HM was acute myeloid leukaemia (AML) (48.4%), and the most common comorbidities were hypertension (20.5%) and diabetes mellitus (12.3%). A total of 164 episodes of BSI were observed in 122 patients, with Gram-negative bacteria isolated in the majority of these episodes (56.7%). In 108 episodes (65.9%), the source of BSIs could not be identified. The most frequent source of infection was central venous catheters (23.2%) (Table 1).

**Table 1:** The clinical and epidemiological characteristics of the patients and BSI episodes in FN

	*n*	(%)
**Gender**		
Male	66	54.1
Female	56	45.9
**Underlying malignancy**		
Acute myeloid leukaemia	59	48.4
Acute lymphoblastic leukaemia	26	21.3
Non-Hodgkin lymphoma	26	21.3
Multiple myeloma	5	4.1
Chronic myeloid leukaemia	2	1.6
Chronic lymphoblastic leukaemia	1	0.8
Others	3	2.5
Total	122	100
**Antimicrobial prophylaxis**		
Antibacterial prophylaxis	40	24.4
Antifungal prophylaxis	78	47.6
**Source of infection**		
Catheter-related BSIs	38	23.2
Pneumonia	6	3.7
Skin abscess	4	2.4
Perianal abscess	3	1.8
Urinary tract infection	2	1.2
Intraabdominal infection	2	1.2
Liver abscess	1	0.6
Source not identified	108	65.9
**Type of microorganism grown**		
Gram-negative	93	56.7
Gram-positive	45	27.4
Fungal	16	9.8
Polymicrobial	10	6.1
Total	164	100
**Neutropenic days (mean** ± S.D.)		
Neutrophil counts < 500 cells/mm^3^	19.35 ± 23.99	
Neutrophil counts < 100 cells/mm^3^	14.58 ± 21.25	

BSI: Bloodstream infection; FN: Febrile neutropenia; HM: Hematological malignancies. S.D.: Standard deviation

**Fig. 1:**
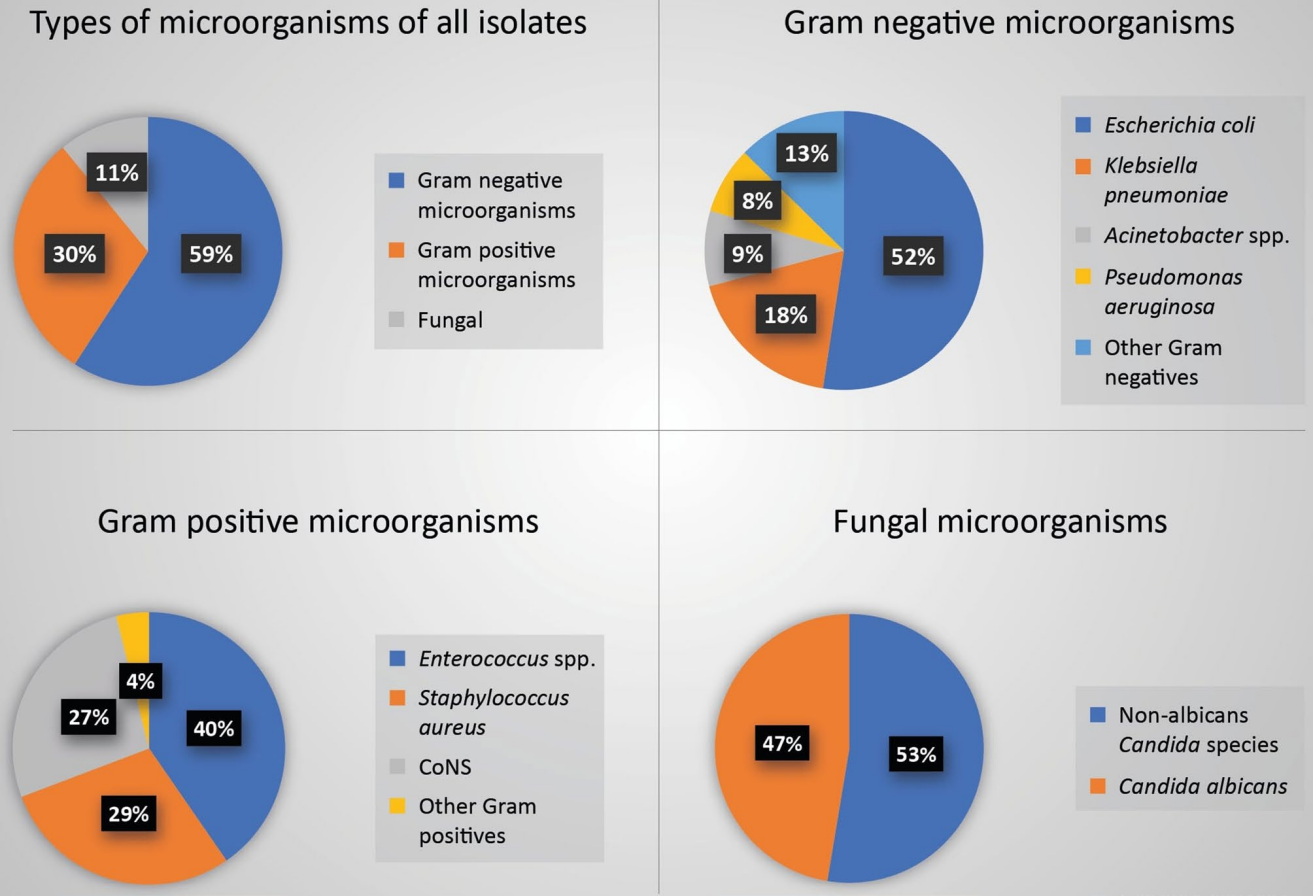
The causative microorganisms of Bloodstream infections. Other Gram-negative bacteria include *Stenotrophomonas maltophilia*, *Enterobacter* spp., *Serratia* spp., *Proteus mirabilis*,* Morganella morganii*,* Salmonella* spp., and *Sphingomonas paucimobilis*. Other Gram-positive bacteria include *Streptococcus pneumoniae* and alpha-hemolytic streptococci. Non-albicans *Candida* species include *Candida krusei*, *Candida glabrata*,* Candida lipolytica*,* Candida tropicalis*, and *Candida parapsilosis. *CoNS: Coagulase negative staphylococci

A total of 174 microorganisms were isolated during FN episodes, including 103 (59.2%) Gram-negative bacteria, of which *Escherichia coli* was the most common pathogen (*n* = 54, 52.4% of Gram-negative isolates). Gram-positive bacteria accounted for 52 (29.9%) isolates, predominantly *Enterococcus* spp. (*n* = 21, 40.4% of Gram-positive isolates), while 19 (10.9%) isolates were fungi, most frequently non-albicans *Candida* species (*n* = 10, 52.6% of fungal isolates) (Fig. 1). A total of 20 microorganisms were isolated in 10 polymicrobial episodes. Of these, 10 (50.0%) were identified as Gram-negative bacteria, 7 (35.0%) as Gram-positive bacteria, and 3 (15.0%) as fungi.

A year-by-year examination of microbial species isolated from blood cultures demonstrated a notable predominance of Gram-negative microorganisms and a relatively limited occurrence of fungal growth. In all years, *E. coli* was more frequently observed among Gram-negative bacterial growths, whereas enterococci were more commonly identified among Gram-positive growths.

Half of the *E. coli* isolates were ESBL-positive. Carbapenem resistance was found in 13.0% of *E. coli isolates*. Among *Klebsiella pneumoniae* isolates, 52.6% were ESBL-positive and carbapenem resistance was observed in 21.1%. Carbapenem resistance was more frequent in *Acinetobacter* spp. and *Pseudomonas aeruginosa* strains (44.4% and 37.5%, respectively). Colistin susceptibility testing was performed selectively in ESBL-positive and/or carbapenem-resistant Gram-negative isolates and was therefore not available for all Gram-negative bacteria. Colistin resistance was detected in three isolates (two *K. pneumoniae* and one *Acinetobacter* spp.).

Among Gram-positive organisms, methicillin resistance was found in coagulase-negative staphylococci (CoNS) (78.6%) and *Staphylococcus aureus* (40.0%). Ampicillin resistance among enterococci was frequent (71.4% overall) and was predominantly observed in *Enterococcus faecium*, in which all isolates were ampicillin-resistant (100%), whereas ampicillin resistance was less common in *Enterococcus faecalis* (20.0%) and other *Enterococcus* spp. (40.0%). Vancomycin resistance was detected in *E. faecium* isolates (27.3%), while no vancomycin resistance was observed in *E. faecalis*, other *Enterococcus* spp., or staphylococcal isolates.

Regarding fungal isolates, fluconazole resistance was not observed in *C. albicans;* however, resistance to fluconazole was identified in 42.9% of non-albicans *Candida* species (Table 2).

**Table 2:** Antimicrobial resistance profiles of the most frequent microorganisms

Gram-negative	ESBL (+) *n* (%)	Carbapenem resistance *n* (%)	Colistin resistance *n* (%)
*E. coli* (*n* = 54)	27 (50)	7 (13)	0 (0)
*K. pneumoniae.* (*n* = 19)	10 (52.6)	4 (21.1)	2 (16.7)
*P. aeruginosa* (*n* = 8)	N/A	3 (37.5)	0 (0)
*Acinetobacter* spp. (*n* = 9)	N/A	4 (44.4)	1 (14.3)^a^
Gram-positive	**Methicillin** **resistance n (%)**	**Ampicillin** **resistance n (%)**	**Vancomycin** **resistance n (%)**
*S. aureus* (*n* = 15)	6 (40)	N/A	0 (0)
CoNS (*n* = 14)	11 (78.6)	N/A	0 (0)
*Enterococcus faecium* (*n* = 11)	N/A	11 (100)	3 (27.3)
*Enterococcus faecalis* (*n* = 5)	N/A	2 (20)	0 (0)
*Enterococcus* spp. (*n* = 5)	N/A	2 (40)	0 (0)
Fungi	**Fluconazole** **resistance n (%)**		
*C. albicans* (*n* = 9)	0 (0)		
non-albicans *Candida* spp. (*n* = 10)	3 (42.9)^b^		

^a^ Colistin susceptibility was tested in 7 *Acinetobacter* spp. strains

^b^ Fluconazole susceptibility was tested in 7 non-albicans *Candida* species

ESBL: Extended spectrum beta-lactamase, N/A: Not applicable, CoNS: Coagulase negative staphylococci

**Fig. 2:**
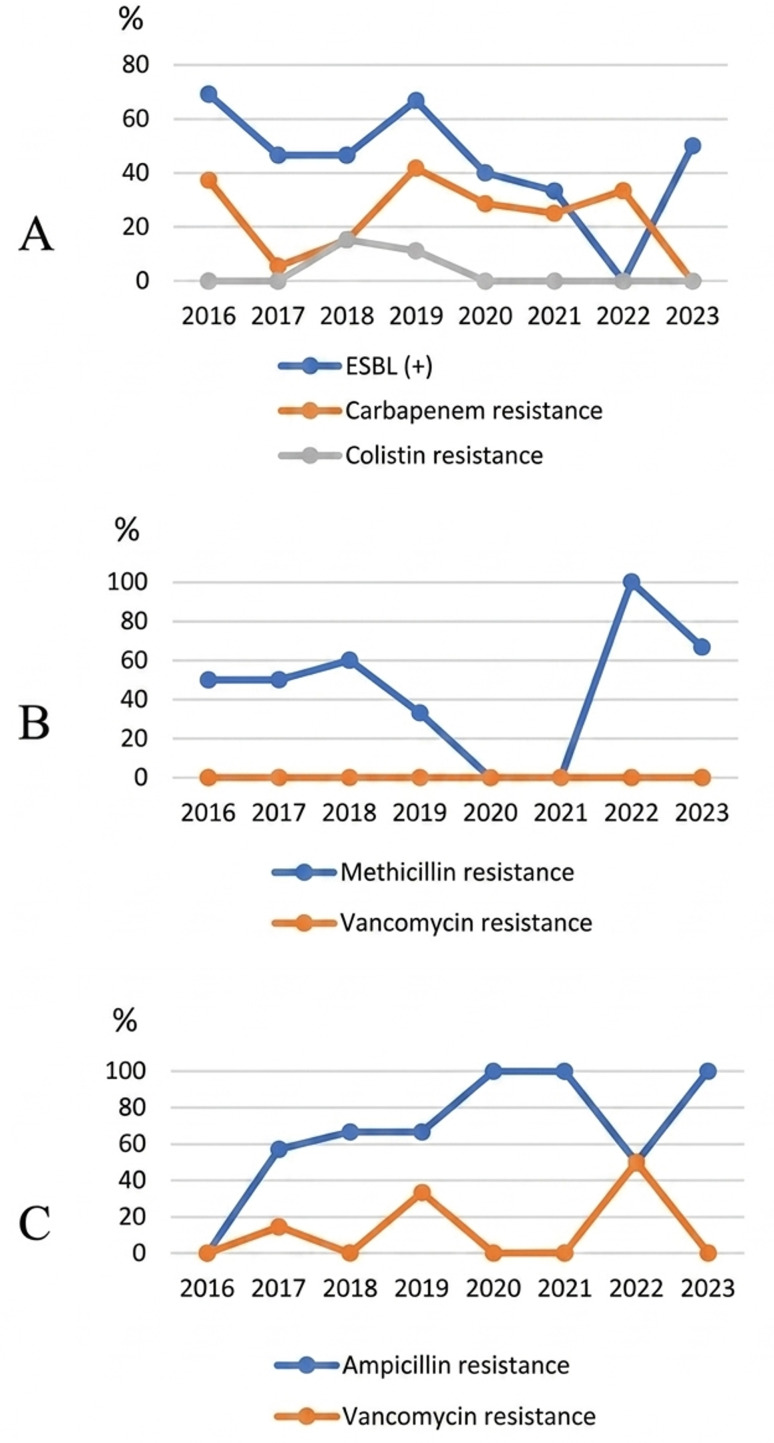
Antimicrobial resistance trends of bloodstream infection isolates over time. **(A)** ESBL-positive, carbapenem-resistant, and colistin-resistant Gram-negative bacteria. **(B)** Methicillin and vancomycin resistance among staphylococci, including coagulase-negative staphylococci and *S. aureus. ***(C)** Ampicillin and vancomycin resistance among *Enterococcus* spp. ESBL: Extended spectrum beta-lactamase

Except for 2018 and 2019, colistin resistance was not observed in Gram-negative bacilli. It is noteworthy that in 2016 and 2019, ESBL positivity and carbapenem resistance were high among Gram-negative bacteria. Furthermore, there has been an increase in ampicillin resistance among enterococci and in methicillin resistance among staphylococci in recent years. The resistance profiles of the microorganisms over the years are shown in Fig. 2.

Antibacterial prophylaxis was administered in 40 (24.4%) episodes and consisted of quinolone-based regimens, most commonly ciprofloxacin (*n* = 29) and levofloxacin (*n* = 11). Antifungal prophylaxis was recorded in 78 (47.6%) episodes. No statistically significant differences were observed between episodes with and without antibacterial prophylaxis in terms of antimicrobial resistance patterns, including ESBL positivity (55.0% with antibacterial prophylaxis vs. 48.1% without prophylaxis) and carbapenem resistance (9.1% vs. 24.7%, respectively). Antibacterial prophylaxis was also not associated with 30-day ICU admission (42.5% vs. 31.5%, *p* = 0.200) or 30-day mortality (37.5% vs. 29.0%, respectively; *p* = 0.314).

Piperacillin-tazobactam was the most frequently initiated empirically therapy, used in 39.0% of BSI episodes, followed by cefepime (15.9%), meropenem (15.2%), and meropenem plus vancomycin (12.8%). Based on antimicrobial susceptibility testing, 50.6% of empirically initiated treatments were appropriate.

Regarding clinical outcomes, ICU admission was required in 56 of 164 episodes (34.1%), and all-cause mortality occurred in 51 episodes (31.1%). The highest ICU admission rates and mortality were observed among BSIs caused by *Stenotrophomonas maltophilia* and *C. albicans* isolates; however, these findings were not statistically significant. The comparison of ICU need and mortality rates by BSI episode type and BSI causative microorganism is shown in Table 3. For BSIs caused by ESBL-positive strains and carbapenem-resistant Gram-negative bacteria, the need for ICU (OR:3.63, *p* = 0.016, and OR:3.42, *p* = 0.02, respectively), and death rates (OR:3.44, *p* = 0.027, and OR:4.30, *p* = 0.006, respectively) were statistically higher. The ICU admissions and mortality rates by microorganism resistance status are shown in Table 4. Additionally, appropriate empirical therapy was significantly associated with reduced ICU admission (24.1% vs. 44.4%, *p* = 0.006) and reduced 30-day mortality (21.7% vs. 40.7%, *p* = 0.008).

**Table 3:** ICU admissions and mortality rates according to BSIs episode types and causative microorganisms

	ICU needed	No need for ICU	*p*	OR	Death	Alive	*p*	OR
	*n* (%)	*n* (%)			*n* (%)	*n* (%)		
Gram-negative episodes (*n* = 93)	32 (34.4)	61 (65.6)	0.674	1.16	29 (31.2)	64 (68.8)	0.825	1.082
* E. coli*	18 (36)	32 (64)	0.598	0.826	15 (30)	35 (70)	0.923	1.037
* Klebsiella* spp.	5 (33.3)	10 (66.7)	0.985	0.989	5 (33.3)	10 (66.7)	0.803	0.866
* P. aeruginosa*	3 (37.5)	5 (62.5)	0.787	0.816	3 (37.5)	5 (62.5)	0.66	0.719
* Acinetobacter* spp.	3 (33.3)	6 (66.7)	0.989	0.99	3 (33.3)	6 (66.7)	0.85	0.871
* S. maltophilia*	3 (75)	1 (25)	0.071	0.157	3 (75)	1 (25)	0.053	0.138
Gram-positive episodes (*n* = 45)	11 (24.4)	34 (75.6)	0.142	0.558	11 (24.4)	34 (75.6)	0.293	0.656
*S. aureus*	4 (30.8)	9 (69.2)	0.851	1.125	4 (30.8)	9 (69.2)	0.984	0.987
CoNS	2 (15.4)	11 (84.6)	0.156	2.929	2 (15.4)	11 (84.6)	0.216	2.578
* Enterococcus* spp.	5 (27.8)	13 (72.2)	0.609	1.329	5 (27.8)	13 (72.2)	0.788	1.162
Fungal episodes (*n* = 16)	8 (50)	8 (50)	0.13	2.298	7 (43.8)	9 (56.2)	0.225	1.906
* C. albicans*	5 (62.5)	3 (37.5)	0.07	0.276	4 (50)	4 (50.0)	0.219	0.417
non-albicans *Candida* species	3 (37.5)	5 (62.5)	0.787	0.816	3 (37.5)	5 (62.5)	0.66	0.719
Polymicrobial episodes (*n* = 10)	5 (50)	5 (50)	0.275	0.495	4 (40)	6 (60)	0.53	0.659
Total (*n* = 164)	56	108			51	113		

ICU: Intensive care unit; BSI: Bloodstream infection

In the chi-square analyses comparing clinical outcomes between specific microorganisms, only monomicrobial isolates were included. Polymicrobial episodes were evaluated as a separate distinct category

**Table 4:** ICU admissions and mortality rates according to antimicrobial resistance profiles of microorganisms

	ICU needed	No need for ICU	*P*	OR	Death	Alive	*p*	OR
	*n* (%)	*n* (%)			*n* (%)	*n* (%)		
**ESBL**								
Presence	16 (48.5)	17 (51.5)	**0.016**	3.63	14 (42.4)	19 (57.6)	**0.027**	3.439
Absence	7 (20.6)	27 (79.4)			6 (17.6)	28 (82.4)		
**Carbapenem resistance**								
Presence	10 (55.6)	8 (44.4)	**0.020**	3.42	10 (55.6)	8 (44.4)	**0.006**	4.297
Absence	19 (26.8)	52 (73.2)			16 (22.5)	55 (77.5)		
**Colistin resistance**								
Presence	17 (39.5)	26 (60.5)	0.356	0.33	15 (34.9)	28 (65.1)	0.27	0.268
Absence	2 (66.7)	1 (33.3)			2 (66.7)	1 (33.3)		
**Methicillin resistance**								
Presence	8 (18.2)	9 (81.8)	0.612	0.61	8 (18.2)	9 (81.8)	0.612	0.611
Absence	4 (26.7)	11 (73.3)			4 (26.7)	11 (73.3)		
**Ampicillin resistance (for** ***Enterococcus*** **spp.)**								
Presence	5 (38.5)	8 (61.5)	--	--	5 (38.5)	8 (61.5)	--	--
Absence	0 (0)	5 (100)			0 (0)	5 (100)		
**Vancomycin resistance (for** ***Enterococcus*** **spp.)**								
Presence	0 (0)	2 (100.0)	--	--	0 (0)	2 (100)	--	--
Absence	5 (31.3)	11 (68.8)			5 (31.3)	11 (68.8)		
**Fluconazole resistance**								
Presence	5 (55.6)	4 (44.4)	--	--	5 (55.6)	4 (44.4)	--	--
Absence	0 (0)	3 (100)			0 (0)	3 (100)		

ICU: Intensive care unit; ESBL: Extended spectrum beta-lactamase

In a secondary analysis, ICU admission and mortality rates were compared between initial and recurrent BSI episodes. ICU admission occurred in 40.5% of recurrent episodes and 32.0% of initial episodes, with no statistically significant difference between groups (*p* = 0.316). Similarly, mortality rates did not differ significantly between recurrent and initial episodes (33.3% vs. 30.3%, *p* = 0.717). Primary disease status was categorized as new diagnosis, relapse, refractory disease, and other disease states. Mortality rates were 28.9% in newly diagnosed patients, 35.3% in relapsed cases, 27.6% in refractory disease, and 32.9% in other disease states, with no statistically significant difference between groups (*p* = 0.915). Similarly, ICU admission rates were 33.3%, 41.2%, 27.6%, and 35.6% across these groups, respectively, with no statistically significant difference (*p* = 0.798). Concomitant infections were infrequent in the study population. CMV viremia was detected in 12 patients (7.3%), *Clostridioides difficile* infection in 10 patients (6.1%), and invasive mold infection in 7 patients (4.3%). Mortality rates were similar between patients with and without CMV viremia (33.3% vs. 30.9%, *p* = 1.000), *C. difficile* infection (20.0% vs. 31.8%, *p* = 0.510), and invasive mold infection (42.9% vs. 30.6%, *p* = 0.678). ICU admission rates were also comparable across these groups (*p* > 0.05 for all comparisons). Due to the limited number of cases, no statistically robust comparisons or adjusted analyses were performed for these variables.

Multivariate logistic regression analyses were performed to evaluate the independent association between antimicrobial resistance phenotypes and clinical outcomes, restricted to first episodes of Gram-negative BSIs (Table 5). ESBL positivity and carbapenem resistance were evaluated in separate models. All models were adjusted for age, sex, underlying hematological malignancy (AML vs. other), and empirical therapy appropriateness. After adjustment, carbapenem resistance remained independently associated with both 30-day ICU admission (adjusted OR: 7.56, 95% CI: 1.30–43.94, *p* = 0.027) and 30-day mortality (adjusted OR: 15.48, 95% CI: 2.08–115.21, *p* = 0.007). In contrast, ESBL positivity was not independently associated with ICU admission (adjusted OR: 3.81, 95% CI: 0.73–19.90, *p* = 0.120) or 30-day mortality (adjusted OR: 5.00, 95% CI: 0.80–31.23, *p* = 0.085). Empirical therapy appropriateness was not independently associated with ICU admission or mortality in multivariable models. Male sex was associated with adverse outcomes in some models, whereas underlying AML was independently associated with ICU admission and mortality in carbapenem resistance models.

**Table 5:** Multivariate logistic regression analyses for ICU admission and 30-day mortality in first Gram-negative BSI episodes

Outcome	Resistance phenotype	*N*	Adjusted OR	95% CI	*p*-value
30-day ICU admission	ESBL-positive	53	3.81	0.73–19.90	0.120
30-day ICU admission	Carbapenem-resistant	72	**7.56**	**1.30-43.94**	**0.027**
30-day mortality	ESBL-positive	53	5.00	0.80-31.23	0.085
30-day mortality	Carbapenem-resistant	72	**15.48**	**2.08-115.21**	**0.007**

BSI: Bloodstream infection; ICU: Intensive care unit; ESBL: Extended spectrum beta-lactamase

All models were adjusted for age, sex, underlying hematological malignancy (AML vs. other), and empirical therapy appropriateness. Analyses were restricted to first Gram-negative BSI episodes with available antimicrobial susceptibility data. ESBL positivity and carbapenem resistance were evaluated in separate models. The multivariate models demonstrated acceptable explanatory power, with Nagelkerke R² values ranging from approximately 0.25 to 0.35

## Discussion

Although recent advances have improved the treatment and supportive care of patients with malignancies, resulting in improved long-term survival rates, infections (particularly BSIs) remain the most common and crucial complication associated with chemotherapy [8]. The rising prevalence of multidrug-resistant microorganisms is an important contributor to mortality [9]. In this study, Gram-negative bacteria—most commonly *Escherichia coli*—were the predominant BSI pathogens. High rates of ESBL production and carbapenem resistance were observed. While ESBL production was not independently associated with adverse outcomes after adjustment, carbapenem resistance remained independently associated with both 30-day ICU admission and all-cause mortality. These findings suggest that not all resistance mechanisms have the same clinical impact, and that carbapenem resistance may represent a more direct determinant of poor prognosis compared to ESBL production.

Similar to our study, studies conducted in the same patient group have shown that Gram-negative bacteria, including *E. coli*, are more frequently isolated in BSIs [10–12]. However, in the studies conducted by Carvalho et al. [13] and Kwon et al. [14], the most frequently occurring type of growth microorganism was Gram-positive bacteria. Although the distribution of Gram-positive organisms varies across studies, CoNS are most frequently reported as the predominant Gram-positive isolates [15–18]. In our study, isolates of CoNS that grew in two separate blood cultures were excluded from the analysis due to not being accepted as causative agents. The relatively low prevalence of CoNS isolates in our study may be attributable to this factor. The overall prevalence of fungi among all microorganisms isolated in our study was 10.9%, whereas other studies have reported lower rates (0% to 7.7%) [11, 13, 19]. Studies in which blood cultures were analysed during FN episodes in patients with HMs reported that the rate of polymicrobial episodes ranged from 6.5% to 17.3% [13, 18, 20]. The observed rate in our study was 6.1%, lower than rates reported in other studies.

ESBL positivity rate was 49.3% among all Gram-negative bacteria, 50% among *E. coli* strains, and 52.6% among *K. pneumoniae* strains in our study. The ESBL positivity rate in *E. coli* strains has been reported to range from 7.1% to 39.9% in other studies conducted on similar patient groups [13, 14, 18]. In addition, the ESBL positivity rate among *K. pneumoniae* strains was reported to range from 6.9% to 48.3% [10, 17, 20]. In our study, carbapenem resistance was found in 20.2% of 99 Gram-negative isolates. The prevalence of carbapenem resistance in *E. coli*,* K. pneumoniae*,* P. aeruginosa* and *Acinetobacter* spp. was 13.0%, 21.1%, 37.5% and 44.4%, respectively. The carbapenem resistance rates of strains isolated from blood cultures in FN episodes of patients with HM were reported to range from 0% to 22% for *E. coli* [10, 18, 20], from 0% to 34.9% for *K. pneumoniae* [13, 14, 21], from 9.1% to 50% for *P. aeruginosa* [13, 17, 19] and from 47.2% to 100% for *Acinetobacter* spp [10, 17, 20]. Notably, carbapenem resistance rates were not low in *E. coli*,* K. pneumoniae*,* P. aeruginosa* and *Acinetobacter* spp. strains frequently isolated during FN episodes. While these international cohorts provide a global framework for BSI trends, it is important to note that regional variations in resistance epidemiology limit the direct clinical applicability of these comparisons to our local setting. Compared with previously published data from Türkiye [17, 20], the ESBL and carbapenem resistance rates observed in our cohort are at the upper end of the reported range, further highlighting the clinical relevance of local resistance patterns when selecting empirical antimicrobial therapy in febrile neutropenic patients. These findings underscore the importance of close monitoring of carbapenem resistance among Gram-negative bacteria and considering local resistance data in empirical antibiotic decision-making for high-risk patients.

In our study, colistin susceptibility was investigated in 52 isolates and colistin resistance was detected in two *K. pneumoniae* and one *Acinetobacter* spp. isolates, which represents a prevalence of 5.8% and is consistent with other studies in the literature [13, 20, 21]. The low resistance rates to colistin, which is an important alternative treatment for carbapenem-resistant bacterial infections, is a positive situation. In our study, the methicillin resistance rate was 78.6% for CoNS and 40% for *S. aureus* strains. The prevalence of methicillin resistance in CoNS isolated from blood cultures during FN episodes in patients with HM has been reported to be 75% and above in similar studies [10, 14, 17]. However, the rates of methicillin resistance in *S. aureus* strains have been reported to range from 19.2% to 63.6% [10, 20, 21]. In our study, the rates of ampicillin and vancomycin resistance among enterococci were 71.4% and 14.3%, respectively. Other similar studies have reported ampicillin resistance ranging from 71.8% to 81.8% [10, 17, 21] and vancomycin resistance ranging from 0.0% to 45.5% [10, 14, 22].

When the antibiotic susceptibility results for the isolates in this study were evaluated, 50.6% of empirically initiated antibiotic treatments were appropriate. The appropriateness of empirical therapy reported in previous studies ranged from 48.4% to 75.3% [11, 20–22]. Given the low appropriateness of empirical treatment at our hospital, local empirical treatment protocols may benefit from periodic review.

In our study, 33.9% of FN episodes required ICU admission, and the 30-day all-cause mortality rate was 30.9%, both of which are higher than those reported in several previous studies [10, 11, 14, 18, 22]. While some studies have reported higher mortality in polymicrobial or Gram-negative FN episodes [14, 18, 21], we did not observe a significant association between microorganism type and mortality in our cohort.

Similar to our study results, Trecarichi et al. [21] observed a statistically significant increase in mortality among BSIs caused by *Enterobacteriaceae* resistant to 3rd-generation cephalosporins and by carbapenem-resistant *K. pneumoniae* strains. In the study by Kara Ali et al. it was shown that ESBL positivity, carbapenem resistance and colistin resistance were found statistically associated with mortality. In contrast to Gram-negative episodes, the presence of antimicrobial resistance in FN episodes caused by Gram-positive isolates was not associated with mortality in our study and the study by Kara Ali et al. [20].

These findings should be interpreted with caution. Residual confounding is an inherent limitation of this study. Although we adjusted for empirical therapy appropriateness, this variable is closely linked to both antimicrobial resistance and underlying disease severity. Consequently, confounding by indication and severity may have influenced the observed associations, and the independent effects of these factors cannot be fully disentangled. Notably, the lack of an independent association between empirical therapy appropriateness and outcomes after adjustment may reflect this complex interplay, rather than a true absence of effect. Mortality in this study was defined as all-cause 30-day mortality. Therefore, deaths may not be directly attributable to BSIs or antimicrobial resistance alone. These findings should be interpreted with caution and should not be considered causal, as other clinical factors may have contributed to mortality.

Our study had several limitations. First, this single-center retrospective cohort study may limit the generalizability of the findings. Second, the primary origin of BSI may not have been assessed optimally due to limited patient data. Third, the crude mortality rate was calculated; however, the mortality attributable to infection could not be calculated because data on comorbidities were unavailable. Fourth, a statistical analysis of year-to-year trends in antimicrobial susceptibility was not performed because the number of isolates per year was limited. Finally, performance status scores were not systematically recorded in the medical records and therefore could not be included in the analyses.

## Conclusions

In this single-center cohort of patients with HM and FN, BSIs caused by ESBL-producing and carbapenem-resistant Gram-negative bacteria were common. However, only carbapenem resistance was independently associated with adverse clinical outcomes. These findings highlight the importance of continuous local surveillance of antimicrobial resistance patterns to inform empirical treatment decisions in similar high-risk clinical settings.

## Data Availability

The datasets analysed during the current study are available from the corresponding author on reasonable request.

## Abbreviations

BSI: Bloodstream infection
CoNS: Coagulase negative staphylococci
ESBL: Extended spectrum beta-lactamase
FN: Febrile neutropenia
HM: Hematological malignancies
ICU: Intensive care unit

## References

[1] Rosa RG, Goldani LZ. Cohort study of the impact of time to antibiotic administration on mortality in patients with febrile neutropenia. Antimicrob Agents Chemother. 2014;58(7):3799–803. 10.1128/AAC.02561-14. 24752269 10.1128/AAC.02561-14 PMC4068526

[2] Viscoli C, Varnier O, Machetti M. Infections in patients with febrile neutropenia: epidemiology, microbiology, and risk stratification. Clin Infect Dis. 2005;40(Suppl 4):S240–5. 10.1086/427329. 15768329 10.1086/427329

[3] Danai PA, Moss M, Mannino DM, Martin GS. The epidemiology of sepsis in patients with malignancy. Chest. 2006;129(6):1432–40. 10.1378/chest.129.6.1432. 16778259 10.1378/chest.129.6.1432

[4] Freelander I, Samarasekara H. Bloodstream infections in haematology/oncology patients: a 5-year retrospective data analysis. Pathology. 2024;56:S121. 10.1016/j.pathol.2023.12.398.

[5] Jacob LA, Lakshmaiah KC, Govindbabu K, Suresh TM, Lokanatha D, Sinha M, et al. Clinical and microbiological profile of febrile neutropenia in solid tumors and hematological malignancies at a tertiary cancer care center in South India. Indian J Cancer. 2014;51(4):464–8. 10.4103/0019-509X.175330. 26842163 10.4103/0019-509X.175330

[6] Taplitz RA, Kennedy EB, Bow EJ, Crews J, Gleason C, Hawley DK, et al. Outpatient Management of Fever and Neutropenia in Adults Treated for Malignancy: American Society of Clinical Oncology and Infectious Diseases Society of America Clinical Practice Guideline Update. J Clin Oncol. 2018;36(14):1443–53. 10.1200/JCO.2017.77.6211. 29461916 10.1200/JCO.2017.77.6211

[7] EUCAST. Clinical breakpoints and dosing of antibiotics [Internet]. Available from: https://www.eucast.org/clinical_breakpoints. Accessed in 2025 (Jan 2).

[8] Wisplinghoff H, Seifert H, Wenzel RP, Edmond MB. Current trends in the epidemiology of nosocomial bloodstream infections in patients with hematological malignancies and solid neoplasms in hospitals in the United States. Clin Infect Dis. 2003;36(9):1103–10. 10.1086/374339. 12715303 10.1086/374339

[9] Paul M, Bhatia M, Rekha US, Diksha BJ, Gupta P. Microbiological profile of bloodstream infections in febrile neutropenic patients at a tertiary care teaching hospital in Rishikesh, Uttarakhand. J Lab Physicians. 2020;12(2):147–153. 10.1055/s-0040-1716661 10.1055/s-0040-1716661 PMC7467830 32905287

[10] Choi H, Ahn H, Lee R, Cho SY, Lee DG. Bloodstream Infections in Patients with Hematologic Diseases: Causative Organisms and Factors Associated with Resistance. Infect Chemother. 2022;54(2):340–52. 10.3947/ic.2022.0069. 35794719 10.3947/ic.2022.0069 PMC9259903

[11] Tang Y, Cheng Q, Yang Q, Liu J, Zhang D, Cao W, et al. Prognostic factors and scoring model of hematological malignancies patients with bloodstream infections. Infection. 2018;46(4):513–21. 10.1007/s15010-018-1151-3. 29767394 10.1007/s15010-018-1151-3

[12] Gedik H. Antibiotic resistance status and its costs in hematological patients: A two-year analysis. Casp J Intern Med. 2017;8(4):276–81. 10.22088/cjim.8.4.276. 10.22088/cjim.8.4.276 PMC5686306 29201318

[13] Carvalho AS, Lagana D, Catford J, Shaw D, Bak N. Bloodstream infections in neutropenic patients with haematological malignancies. Infect Dis Health. 2020;25(1):22–9. 10.1016/j.idh.2019.08.006. 31586572 10.1016/j.idh.2019.08.006

[14] Kwon JC, Kim SH, Choi JK, Cho SY, Park YJ, Park SH, et al. Epidemiology and clinical features of bloodstream infections in hematology wards: one year experience at the catholic blood and marrow transplantation center. Infect Chemother. 2013;45(1):51–61. 10.3947/ic.2013.45.1.51. 24265950 10.3947/ic.2013.45.1.51 PMC3780938

[15] Gudiol C, Bodro M, Simonetti A, Tubau F, González-Barca E, Cisnal M, et al. Changing aetiology, clinical features, antimicrobial resistance, and outcomes of bloodstream infection in neutropenic cancer patients. Clin Microbiol Infect. 2013;19(5):474–9. 10.1111/j.1469-0691.2012.03879.x. 22524597 10.1111/j.1469-0691.2012.03879.x

[16] Hamidi AA, Basaran S, Cagatay AA, Ozsut H, Atay K, Avsar N, et al. Organisms Isolated from Blood Cultures, Their Antimicrobial Susceptibilities and Patient Characteristics in Patients with Febrile Neutropenia. Klimik. 2009;22:88–91.

[17] Kara Ö, Zarakolu P, Aşçioğlu S, Etgül S, Uz B, Büyükaşik Y, et al. Epidemiology and emerging resistance in bacterial bloodstream infections in patients with hematologic malignancies. Infect Dis (Lond). 2015;47(10):686–93. 10.3109/23744235.2015.1051105. 26024284 10.3109/23744235.2015.1051105

[18] El Omri H, Padmanabhan R, Taha RY, Kassem N, Elsabah H, Ellahie AY, et al. Dissecting bloodstream infections in febrile neutropenic patients with hematological malignancies, a decade-long single center retrospective observational study (2009–2019). J Infect Public Health. 2024;17(1):152–62. 10.1016/j.jiph.2023.11.017. 38029491 10.1016/j.jiph.2023.11.017

[19] Ghosh I, Raina V, Kumar L, Sharma A, Bakhshi S, Thulkar S, et al. Profile of infections and outcome in high-risk febrile neutropenia: experience from a tertiary care cancer center in India. Med Oncol. 2012;29(2):1354–60. 10.1007/s12032-011-9858-3. 21336987 10.1007/s12032-011-9858-3

[20] Kara Ali R, Surme S, Balkan II, Salihoglu A, Sahin Ozdemir M, Ozdemir Y, et al. An eleven-year cohort of bloodstream infections in 552 febrile neutropenic patients: resistance profiles of Gram-negative bacteria as a predictor of mortality. Ann Hematol. 2020;99(8):1925–32. 10.1007/s00277-020-04144-w. 32564194 10.1007/s00277-020-04144-w

[21] Trecarichi EM, Pagano L, Candoni A, Pastore D, Cattaneo C, Fanci R, et al. HeMABIS Registry-SEIFEM Group, Current epidemiology and antimicrobial resistance data for bacterial bloodstream infections in patients with hematologic malignancies: an Italian multicentre prospective survey. Clin Microbiol Infect. 2015;21(4):337–43. 10.1016/j.cmi.2014.11.022. 25595706 10.1016/j.cmi.2014.11.022

[22] Mert D, Ceken S, Iskender G, Iskender D, Merdin A, Duygu F, et al. Epidemiology and mortality in bacterial bloodstream infections in patients with hematologic malignancies. J Infect Dev Ctries. 2019;13(8):727–35. 10.3855/jidc.11457. 32069257 10.3855/jidc.11457

